# cropCSM: designing safe and potent herbicides with graph-based signatures

**DOI:** 10.1093/bib/bbac042

**Published:** 2022-02-24

**Authors:** Douglas E V Pires, Keith A Stubbs, Joshua S Mylne, David B Ascher

**Affiliations:** School of Computing and Information Systems at the University of Melbourne; School of Molecular Sciences at the University of Western Australia; Curtin University and Deputy Director of the Centre for Crop and Disease Management; University of Queensland, and head of Computational Biology and Clinical Informatics at the Baker Institute and Systems

**Keywords:** machine learning, graph-based signatures, herbicides, toxicity

## Abstract

Herbicides have revolutionised weed management, increased crop yields and improved profitability allowing for an increase in worldwide food security. Their widespread use, however, has also led to a rise in resistance and concerns about their environmental impact. Despite the need for potent and safe herbicidal molecules, no herbicide with a new mode of action has reached the market in 30 years. Although development of computational approaches has proven invaluable to guide rational drug discovery pipelines, leading to higher hit rates and lower attrition due to poor toxicity, little has been done in contrast for herbicide design. To fill this gap, we have developed cropCSM, a computational platform to help identify new, potent, nontoxic and environmentally safe herbicides. By using a knowledge-based approach, we identified physicochemical properties and substructures enriched in safe herbicides. By representing the small molecules as a graph, we leveraged these insights to guide the development of predictive models trained and tested on the largest collected data set of molecules with experimentally characterised herbicidal profiles to date (over 4500 compounds). In addition, we developed six new environmental and human toxicity predictors, spanning five different species to assist in molecule prioritisation. cropCSM was able to correctly identify 97% of herbicides currently available commercially, while predicting toxicity profiles with accuracies of up to 92%. We believe cropCSM will be an essential tool for the enrichment of screening libraries and to guide the development of potent and safe herbicides. We have made the method freely available through a user-friendly webserver at http://biosig.unimelb.edu.au/crop_csm.

## Introduction

Herbicides are widely used chemical agents capable of killing or inhibiting growth of unwanted plants, including weeds and grass types, that might compromise crop yields. Over the years, their adoption has revolutionised weed management, increased crop yields and improved profitability allowing for an increase in worldwide food security.

Their widespread use, however, has also led to a rise in resistance [1]. Without appropriate measures to bring herbicides with new modes of action to the market, combined with a concerted global effort at product stewardship and regulation, herbicide resistance could reduce world food production in the coming years by 20–40%, leading to a global food security crisis [2]. Although agricultural practices have improved, decades of success by glyphosate and spiralling costs have stymied herbicide development. Designing potent herbicides is particularly challenging considering toxicity concerns, and currently available and widely used weed control compounds have shown to be potentially harmful for the environment, livestock and human health. Exposure to agrochemicals has been, for instance, shown to be one of the potential drivers to the decline of pollinators worldwide [3]. Despite the need for novel potent and safe herbicidal molecules, no herbicide with a new mode of action has reached the market in 30 years [4].

Developing herbicides, much like pharmaceuticals, involves a careful balance between efficacy and safety. In the pharmaceutical industry, drug development pipelines have tackled these challenges by modelling and optimising these important parameters early in the development process, which has been assisted by the implementation of computational pipelines. This has led, in general, to increased hit rates and decreased attrition due to poor toxicity profiles and, in the process, reduced development time, costs and animal testing [5–10]. Although many computer-aided approaches have proven invaluable for drug development, in contrast little has been done to aid the development of safe and potent agrochemicals. A relevant recent development has been the work of Oršolić *et al*. to establish structure–activity relationships linking herbicides with modes of action and weed selectivity [11].

**Figure 1:**
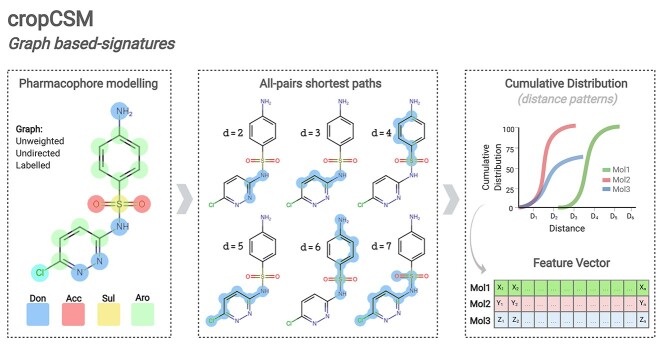
Modelling small molecule activity using graph-based signatures. Small molecules are modelled as unweighted, undirected graphs where nodes represent atoms and edges represent chemical bonds, with atoms labelled via pharmacophore modelling (left panel). All-pairs shortest paths distances (d) are calculated between different label types (middle panel) to represent molecules, their geometry and physicochemical properties as cumulative distributions (right panel, considering different cutoff/distance values).

To fill this gap, here we describe the design of the first computational method to support and guide rational development of safe and potent herbicides, cropCSM, using the largest curated small molecule database of experimentally characterised herbicidal activity to date. Our method uses the concept of graph-based signatures, represented as a cutoff scanning matrix (CSM) (Figure 1), to model small molecule physicochemical properties coupled with machine learning to train and validate predictive tools capable of accurately identifying molecules with potent herbicidal activity, as well as characterise their environmental and human toxicity profiles.

## Materials and methods

cropCSM development was divided into four main steps, including (i) data acquisition, which involved obtaining data relating small molecules to their herbicidal activity as well as human and environmental toxicity profiles; (ii) feature engineering, which involved calculating molecular properties and performing graph-based modelling to extracting relevant information used as evidence in the next steps to (iii) identify what makes up potent and safe herbicides, identifying enriched substructures and qualitatively assessing molecular properties and to (iv) train and test predictive model using supervised learning algorithms.

### Data for herbicidal activity

A dataset of 4513 experimentally characterized, structurally diverse small molecules and their herbicidal activity profiles was obtained from Sukhoverkov *et al*. [12]. These were labelled either as active (997 molecules) or inactive (3516 molecules). They had an average molecular weight of 380 Da and logP of 2.4 (Figure S1). A database of 356 commercial herbicides was also used to evaluate cropCSM [12].

### Data for environmental and human toxicity

We have developed new predictors based on six environmental and human toxicity data sets with experimentally characterised molecules. Environmental toxicity data sets included (a) honey bee (*Apis mellifera*) toxicity, which was composed of 247 toxic and 353 atoxic molecules [13]; (b) avian toxicity, composed of 461 small molecules and their effects on mallard duck (66 toxic and 395 atoxic) [14] and (c) flathead minnow toxicity, with lethal concentration values (LC50) for a diverse set of 554 molecules [15]. Human toxicity data sets included (1) AMES toxicity, with compounds labelled based on their carcinogenic potential (4632 carcinogenic and 3470 not carcinogenic) [16]; (2) oral acute toxicity in rats, denoted as lethal dose (LD50) values for 10 145 compounds [17] and (3) oral chronic toxicity in rats values for 567 compounds [18].

**Figure 2:**
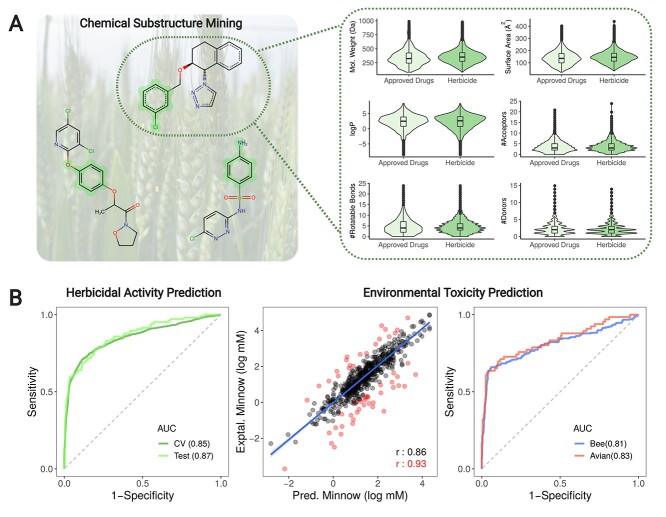
cropCSM: predicting safe and potent herbicides. Using chemical substructure mining, we identified common enriched substructures in compounds with herbicidal activity (**A**-left). Active compounds presented similar molecular properties of approved drugs (**A**-right). Performance of herbicide and environmental-toxicity predictors is shown in (**B**). Our herbicide predictor was able to accurately identify active compounds with AUC > 0.85 on cross-validation and blind test. Three environmental toxicity models have been developed and were capable of successfully measuring minnow toxicity (as a regression task, centre graph) as well as identifying potentially harmful compounds for Bees and Mallard (right-hand side graph). Sensitivity refers to the true positive rate, whereas specificity to the true negative rate.

### Graph-based signatures and feature engineering

Graph modelling is an invaluable tool to model biological entities, including small molecules [19–22]. Over the years we have proposed and developed the concept of graph-based signatures (based on CSM concept [23]) to represent physicochemical and geometrical properties of a range of macromolecules [24–29] and their interactions [30–37]. These have also been successfully adapted to represent small molecules pharmacokinetics, toxicity and bioactivity [38–41]. Figure 1 depicts the main steps involved in feature engineering with graph-based signatures. Small molecules are modelled as unweighted, undirected graphs where nodes represent atoms and edges represent covalent bonds. Via pharmacophore modelling [38], atoms/nodes are labelled based on their physicochemical properties. This atomic graph representation of small molecules accounts for both their shape and composition, from which information is extracted as distance patterns. To do so, all-pairs shortest paths distances are calculated, and molecules are then represented simply as cumulative distribution functions of atom distances. These distances are further categorised based on their respective physicochemical properties (pharmacophores) and converted as a feature vector used as evidence to train and test predictive methods. Complementary physicochemical properties are calculated and included using the RDKit cheminformatics library [42] and included in the feature vector.

### Model selection and validation

Different supervised learning algorithms available on the scikit-learn Python library [43] were assessed with best performing models (Random Forest, *n_estimators =* 300 for herbicide activity). Information on learning algorithms and hyperparameters for remaining models is available as Table S1 and selected based on accuracy, Matthew’s correlation coefficient (MCC) and the area under the Receiver operating characteristic curve (ROC curve) (AUC) for classification tasks and Pearson’s correlation [44] and root mean squared error (RMSE) for regression tasks. Hyperparameter tuning was performed with a grid search method implemented in scikit-learn; however, no significant improvement in performance was observed. Performance was assessed under stratified 10-fold cross-validation (obtained using scikit-learn Python library) [29, 30, 45] as well as using nonredundant blind tests (blind tests were composed of held out sets accounting for 10% of molecules selected at random and used as external validation). A feature selection step was used to reduce dimensionality and improve performance via a stepwise forward greedy selection approach [24, 28–30, 46–48].

**Table 1:** Performance of cropCSM on identifying molecules with herbicidal activity.

**Data set**	**Performance metrics**		
	**Accuracy**	**AUC**	**MCC**
Cross-validation	87%	0.85	0.60
Blind test	87%	0.87	0.59

**Figure 3:**
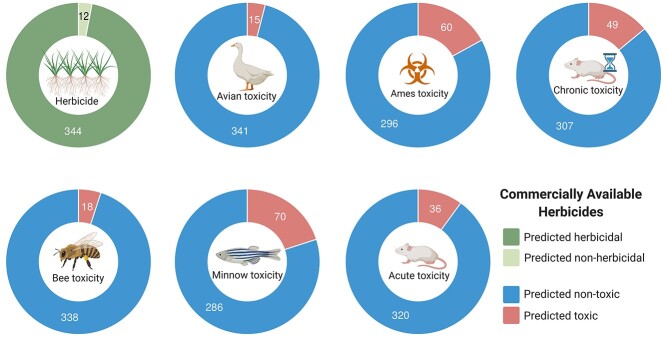
Performance of cropCSM on commercially available herbicides. Our method was able to correctly classify 97% of commercial herbicides (344 out of 356, top-left graph). The figure also shows the proportion of compounds predicted to be environmental or human toxic. Molecules were more frequently predicted as AMES toxic (17%, 60 out of 356) and minnow toxic (20%, 70 out of 356).

### Substructure mining

To identify what makes up a herbicide, molecular substructure mining was employed to identify substructures that were enriched in the herbicidal class and depleted in the nonherbicidal class. For this purpose, the molecular substructure miner (MoSS) [49] tool was used, and different minimum support/frequency cutoffs tested. We considered 5- and 10-fold differences between support of positive and negative classes (1%, 5%, 1–10% and 2–10%).

### Data visualization

t-distributed stochastic neighbor embedding (t-SNE) plots were generated using the R package ‘tsne’ with default parameters.

## Results

### What makes an herbicide? Correlating molecular properties with herbicidal activity

Using experimental information on the herbicidal activity for a collected data set of over 4500 small molecule compounds (22% with herbicidal activity), we investigated what physicochemical properties of the compounds translate to herbicidal activity.

Herbicidal molecules were enriched in saturated carbon chains and benzene substructures, compared with the inactive molecules (Figure 2**A**). The majority (90%) of the active compounds tended to be < 517 Da, have up to 9 acceptors and 4 donors, fewer than 9 rotatable bonds and a logP between −1.7 and 6.1 (Figure S1) (95% <700 Da, 11 rotatable bonds, 11 acceptors, 6 donors and logP between −3.0 to 6.1). This is similar, although slightly more lenient, than the widely used Lipinski’s Rule of Five for orally bioavailable drugs. Interestingly, but consistently, there was no significant distinction in physicochemical properties between herbicides and approved drugs, as illustrated in the t-SNE plot (Figure S2). Compared with all U.S. food and drug administration (FDA)-approved drugs, however, herbicides were enriched in substructures involving chlorine.

Herbicides have been previously compared with pharmaceuticals [10] with our analysis being consistent with previous results across smaller datasets [50–54], which have shown that physicochemical properties of herbicides are similar to orally delivered drugs, although the former tend to be smaller, with fewer proton donors, lower partition coefficient [53, 54].

**Figure 4:**
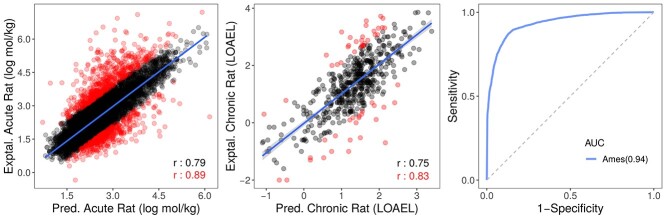
Performance on cross-validation of human-toxicity predictors. Three models have been developed and were capable of accurately measuring acute and chronic rat toxicity (as regression tasks, left and centre graphs) as well as identifying potentially carcinogenic compounds (Ames toxicity as a classification task, right-hand side graph). For regression models, two Pearson’s correlation coefficients are shown: one obtained for the whole data set (in black) and one on 90% of the data, after 10% of outliers or poorly predicted points are removed (in red) for analysis purposes to assess the potential effects of outliers on performance (depicted as red dots).

**Table 2:** Performance of cropCSM on identifying environmentally toxic compounds as a regression task

**Environmental Toxicity**	**Cross-validation**	
	**Pearson’s (*r*)**	**RMSE**
***Minnow toxicity (LC50)***		
cropCSM	0.86	0.74
pkCSM	0.74^*^	0.84
admetSAR	0.57^*^	0.67
***Oral rat chronic toxicity***		
cropCSM	0.75	0.56
pkCSM	0.68^*^	0.74
admetSAR	0.50^*^	0.73
***Oral rat acute toxicity (LD50)***		
cropCSM	0.79	0.62
pkCSM	0.66^*^	0.68
admetSAR	0.61^*^	0.32

^*^Denotes a statistically significant performance difference obtained via a Fisher *r* − to−*z* transformation, by calculating the *z* value, using a threshold of *P* ≤ 0.05 for significance. The table presents performance assessed on cross-validation.

**Table 3:** Performance of cropCSM on identifying environmentally toxic compounds as a classification task

**Environmental toxicity**	**Cross-validation**		
	**Accuracy**	**AUC**	**MCC**
***Honey bee toxicity***			
cropCSM	87%	0.81	0.65
Wang *et al*.	84%	0.84	0.60
***Avian toxicity***			
cropCSM	92%	0.83	0.65
Zhang *et al*.	91%	0.91	NA
***AMES toxicity***			
cropCSM	87%	0.94	0.74
pkCSM	84%	0.91	NA
admetSAR	85%	0.91	NA

The table presents performance assessed on cross-validation. NA: Not available.

These insights were used as a platform to build a supervised machine learning predictive model to identify herbicidal compounds, where the small molecule structure was represented as a graph-based signature, termed CSM, in which the atoms are represented as nodes, and covalent interactions between them as edges [38, 45]. Under stratified 10-fold cross-validation, we were able to correctly identify 82% of the active molecules with an overall accuracy of 87%, area under the ROC curve (AUC) of 0.85 and MCC of 0.60 (Figure 2**B** and Table 1). For compounds over 500 Da, cropCSM achieved an AUC of 0.81, illustrating the robust performance of the approach on larger compounds. A precision-recall curve (PR curve) was also calculated (Figure S3) to investigate the compromises between type I and type II errors. cropCSM achieved a PR AUC of 0.93, demonstrating a well-tuned model, with good performance and balance between type error I and II. When the model was evaluated against a blind test set of 106 active and 345 inactive molecules, we achieved comparable performance (87% accuracy, AUC of 0.87 and MCC of 0.59). This provided confidence that the approach can be generalized and used with unknown sets of putative herbicidal molecules active against a target of interest.

To further validate the cropCSM models and demonstrate their real-world applicability, they were applied to a set of 356 commercial herbicides [12]. Over 97% were correctly identified as herbicidal (Figure 3). Of those that were not, they included the natural fatty acid oleic acid and fragment-like molecules such as dazomet and pentachlorophenol.

**Figure 5:**
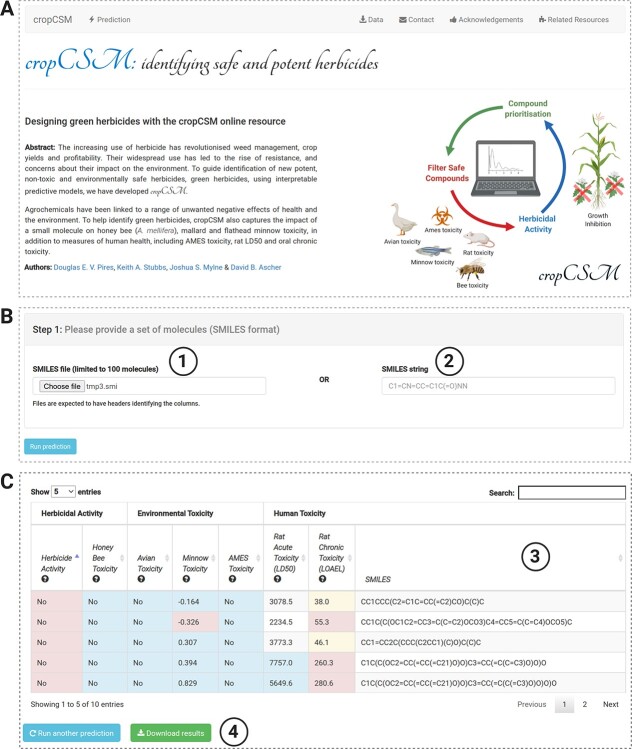
cropCSM web server. (**A**) Depicts the landing page for the resource. By clicking on ‘Prediction’ at the top menu, users are directed to the job submission page (**B**). There users have the options to either provide a set of molecules as a SMILES file (1) or individual molecules as a SMILES string (2). After clicking on ‘Run prediction’, and once calculations are complete, users are redirected to a results page (**C**) where predictions for herbicidal activity as well as toxicity profiles are presented in tabular format (3). Users have the options to download the results (4).

### Predicting environmental toxicity

Agrochemicals have been linked to a range of unwanted negative effects on both health and the environment, including glyphosate-free herbicides such as benzalkonium chloride [55]. To help identify safe herbicides, complementary models were developed to capture the impact of a small molecule on honey bee (*A. mellifera*), mallard (*Anas platyrhynchos*) and flathead minnow (*Pimephales promelas*) toxicity, in addition to measures of human health, including AMES toxicity, rat LD50 and oral chronic toxicity. Although assessing molecular substructures enriched in toxic compounds (based on these datasets), (Figure S4), we identified a prevalence of complex ring structures. Of note, structures rich in chlorine, while enriched in herbicides, were also enriched in compounds that were toxic for mallard and minnow, highlighting a potential inherent difficulty in optimising potency and safety when designing herbicides.

Tables 2 and 3 depict the performance of cropCSM models for identifying toxic molecules as regression and classification tasks, respectively, assessed during cross-validation. Overall, we were also able to identify toxic molecules as classification and regression tasks with accuracies of up to 92% and Pearson’s correlations of up to 0.86, outperforming previous predictive approaches [13, 14, 38, 56], including pkCSM, admetSAR and the works from Wang *et al.* and Zhang *et al*. Regarding environmental toxicity models, the minnow toxicity (LC50) model achieved a Pearson’s correlation of 0.86 (RMSE of 0.74), a performance that increases to 0.93 on 90% of the data (after removing 10% outliers – these are only removed for analysis purposes to assess the potential effects of outliers on performance), whereas honeybee and avian toxicity classifiers achieved AUCs of 0.81 and 0.83 (Figure 2**B**). For human health toxicity predictors, cropCSM achieve Pearson’s correlation coefficients of 0.79 and 0.75 for oral rat acute and chronic toxicity, respectively, and an AUC of 0.94 while predicting AMES compounds (Figure 4).

Using these validated methods, we set out to assess the toxicity profiles of currently commercially available herbicides. The list of 356 commercial herbicides was submitted to the predictors as an independent test. Figure 3 depicts a large proportion of compounds predicted to be environmental or human toxic, with molecules more frequently predicted as either AMES toxic (17%, 60 out of 356) or minnow toxic (20%, 70 out of 356). Interestingly, widely used herbicides glyphosate and glufosinate were predicted as nontoxic to humans. This is interesting, as while glyphosates have been proposed to be carcinogenic, there is growing support that it is the inert products that are responsible for damage to human beings and the environment [57], which would be consistent with the predictions from cropCSM. When grouping commercial herbicides based on their mode of action, a significant proportion of minnow toxic compounds belonged to the class of inhibitors of acetyl CoA carboxylase (14/24, 58% versus 20% overall), AMES toxic compounds were enriched in the class of Inhibitors of cell division (10/28, 36% versus 17% overall) and compounds involved in Rat Chronic toxicity were more frequent in the class of inhibitors of lipid synthesis (7/19, 37% versus 14% overall). These results add credence to the tool to rapidly identify potentially hazardous molecules early in the development process, which has the potential to significantly reduce costs and failure rates.

### The cropCSM web server

The backend of the cropCSM web server was developed using the Python Flask framework version 0.12.3 and the front end using Bootstrap framework version 3.3.7. The system is hosted by a Linux server running Apache. Figure 5**A** depicts the cropCSM web interface. Users can submit molecules to the server either individually as SMILES strings or in batch as SMILES files (Figure 5**B**). Prediction results are displayed in tabular format (Figure 5**C**), which are also made available to download as a comma-separated file.

## Conclusions

Here we described cropCSM, the first free and easy-to-use *in silico* platform dedicated to assist the development of herbicides that are potent, but also nontoxic and environmentally safe. cropCSM is optimally designed to evaluate the herbicide potential and toxicity of small molecular compounds up to 2 kDa, consistent with the training and validation data sets. We employed graph-based signatures to model small molecule physicochemistry of the largest collected data set of molecules with experimentally characterised herbicidal profiles to date and demonstrated their efficacy. We anticipate future iterations of cropCSM that will draw upon larger datasets and as a result will have a higher predictor capability, allowing for a greater increase in accuracy and correlation. We believe cropCSM will be an invaluable tool to assist rational design of new potent herbicidal molecules that are also safe for humans and the environment. The tool has been made available as a user-friendly web interface at https://biosig.unimelb.edu.au/crop_csm.

**Key Points**

- Although very little has been done to assist the designing of potent and safe herbicides computationally, cropCSM fills this gap.
- cropCSM can accurately identify small molecules with potential herbicidal activity as well as characterise their toxicity and safety profiles.
- A web server conveniently provides cropCSM’s models in a free and easy-to-use interface.

## Supplementary Material

cropCSM-Supplementary_bbac042

## Data Availability

Models are freely available at: http://biosig.unimelb.edu.au/crop_csm.

## Notes

**Douglas E. V. Pires** is a Senior Lecturer in Digital Health with the School of Computing and Information Systems at the University of Melbourne and group leader at Bio21 Institute. He is a computer scientist and bioinformatician specialising in machine learning and AI and the development of tools to analyse omics data.

**Keith A. Stubbs** is an Associate Professor and group leader in the School of Molecular Sciences at the University of Western Australia. He is a chemical biologist who has interests in developing chemical tools to study biological questions of interest in the areas of chemical glycobiology, antimicrobials and herbicide discovery.

**Joshua S. Mylne** is a Professor at Curtin University and Deputy Director of the Centre for Crop and Disease Management. He is a plant molecular biologist specialising in protein biochemistry and agrochemicals research, specifically the discovery of herbicides with new modes of action and the in planta metabolism of fungicides.

**David B. Ascher** is Deputy Director of Biotechnology at The University of Queensland, and head of Computational Biology and Clinical Informatics at the Baker Institute and Systems and computational Biology at Bio21 Institute. He is interested in developing and applying computational tools to assist leveraging clinical and omics data for drug discovery and personalised medicine.

## References

[1] Delye C, Jasieniuk M, Le Corre V. Deciphering the evolution of herbicide resistance in weeds. Trends Genet 2013;29:649–58. 23830583 10.1016/j.tig.2013.06.001

[2] Peterson MA, Collavo A, Ovejero R, et al. The challenge of herbicide resistance around the world: a current summary. Pest Manag Sci 2018;74:2246–59. 29222931 10.1002/ps.4821

[3] Potts SG, Biesmeijer JC, Kremen C, et al. Global pollinator declines: trends, impacts and drivers. Trends Ecol Evol 2010;25:345–53. 20188434 10.1016/j.tree.2010.01.007

[4] Davis AS, Frisvold GB. Are herbicides a once in a century method of weed control? Pest Manag Sci 2017;73:2209–20. 28618159 10.1002/ps.4643

[5] Kola I, Landis J. Can the pharmaceutical industry reduce attrition rates? Nat Rev Drug Discov 2004;3:711–6. 15286737 10.1038/nrd1470

[6] Ulrich R, Friend SH. Toxicogenomics and drug discovery: will new technologies help us produce better drugs? Nat Rev Drug Discov 2002;1:84–8. 12119613 10.1038/nrd710

[7] Waring MJ, Arrowsmith J, Leach AR, et al. An analysis of the attrition of drug candidates from four major pharmaceutical companies. Nat Rev Drug Discov 2015;14:475–86. 26091267 10.1038/nrd4609

[8] Segall MD, Barber C. Addressing toxicity risk when designing and selecting compounds in early drug discovery. Drug Discov Today 2014;19:688–93. 24451294 10.1016/j.drudis.2014.01.006

[9] Corral MG, Leroux J, Stubbs KA, et al. Herbicidal properties of antimalarial drugs. Sci Rep 2017;7:45871. 28361906 10.1038/srep45871 PMC5374466

[10] Delaney J, Clarke E, Hughes D, et al. Modern agrochemical research: a missed opportunity for drug discovery? Drug Discov Today 2006;11:839–45. 16935753 10.1016/j.drudis.2006.07.002

[11] Oršolić D, Pehar V, Šmuc T, et al. Comprehensive machine learning based study of the chemical space of herbicides. Sci Rep 2021;11:11479. 34075109 10.1038/s41598-021-90690-w PMC8169684

[12] Sukhoverkov KV, Corral MG, Leroux J, et al. Improved herbicide discovery using physico-chemical rules refined by antimalarial library screening. RSC Advances 2021;11(15):8459–67. 35423398 10.1039/d1ra00914a PMC8695207

[13] Wang F, Yang J-F, Wang M-Y, et al. Graph attention convolutional neural network model for chemical poisoning of honey bees’ prediction. Sci Bull 2020;65:1184–91. 10.1016/j.scib.2020.04.006 36659148

[14] Zhang C, Cheng F, Sun L, et al. In silico prediction of chemical toxicity on avian species using chemical category approaches. Chemosphere 2015;122:280–7. 25532772 10.1016/j.chemosphere.2014.12.001

[15] Weihua L, Yun T. In silico prediction of terrestrial and aquatic toxicities for organic chemicals. Chin J Pest Sci 2010;12(4):477–88.

[16] Xu C, Cheng F, Chen L, et al. In silico prediction of chemical Ames mutagenicity. J Chem Inf Model 2012;52:2840–7. 23030379 10.1021/ci300400a

[17] Zhu H, Martin TM, Ye L, et al. Quantitative structure-activity relationship modeling of rat acute toxicity by oral exposure. Chem Res Toxicol 2009;22:1913–21. 19845371 10.1021/tx900189p PMC2796713

[18] Mazzatorta P, Estevez MD, Coulet M, et al. Modeling oral rat chronic toxicity. J Chem Inf Model 2008;48:1949–54. 18803370 10.1021/ci8001974

[19] Zhang Z, Guan J, Zhou S. FraGAT: a fragment-oriented multi-scale graph attention model for molecular property prediction. Bioinformatics 2021;37:2981–7. 10.1093/bioinformatics/btab195 PMC8479684 33769437

[20] Shen WX, Zeng X, Zhu F, et al. Out-of-the-box deep learning prediction of pharmaceutical properties by broadly learned knowledge-based molecular representations. Nat Mach Intell 2021;3:334–43.

[21] Imrie F, Bradley AR, van der Schaar M, et al. Deep generative models for 3D linker design. J Chem Inf Model 2020;60:1983–95. 32195587 10.1021/acs.jcim.9b01120 PMC7189367

[22] David L, Thakkar A, Mercado R, et al. Molecular representations in AI-driven drug discovery: a review and practical guide. J Chem 2020;12:56. 10.1186/s13321-020-00460-5 PMC7495975 33431035

[23] Pires DE, de Melo-Minardi RC, dos Santos MA, et al. Cutoff scanning matrix (CSM): structural classification and function prediction by protein inter-residue distance patterns. BMC Genomics 2011;12(Suppl 4):S12. 10.1186/1471-2164-12-S4-S12 PMC3287581 22369665

[24] Kaminskas LM, Pires DEV, Ascher DB. dendPoint: a web resource for dendrimer pharmacokinetics investigation and prediction. Sci Rep 2019;9:15465. 31664080 10.1038/s41598-019-51789-3 PMC6820739

[25] Pires DE, Ascher DB, Blundell TL. DUET: a server for predicting effects of mutations on protein stability using an integrated computational approach. Nucleic Acids Res 2014;42:W314–9. 24829462 10.1093/nar/gku411 PMC4086143

[26] Pires DE, Ascher DB, Blundell TL. mCSM: predicting the effects of mutations in proteins using graph-based signatures. Bioinformatics 2014;30:335–42. 24281696 10.1093/bioinformatics/btt691 PMC3904523

[27] Pires DEV, Rodrigues CHM, Ascher DB. mCSM-membrane: predicting the effects of mutations on transmembrane proteins. Nucleic Acids Res 2020;48:W147–53. 32469063 10.1093/nar/gkaa416 PMC7319563

[28] Rodrigues CH, Pires DE, Ascher DB. DynaMut: predicting the impact of mutations on protein conformation, flexibility and stability. Nucleic Acids Res 2018;46:W350–5. 29718330 10.1093/nar/gky300 PMC6031064

[29] Rodrigues CHM, Pires DEV, Ascher DB. DynaMut2: assessing changes in stability and flexibility upon single and multiple point missense mutations. Protein Sci 2021;30:60–9. 32881105 10.1002/pro.3942 PMC7737773

[30] Myung Y, Pires DEV, Ascher DB. mmCSM-AB: guiding rational antibody engineering through multiple point mutations. Nucleic Acids Res 2020;48:W125–31. 32432715 10.1093/nar/gkaa389 PMC7319589

[31] Myung Y, Rodrigues CHM, Ascher DB, et al. mCSM-AB2: guiding rational antibody design using graph-based signatures. Bioinformatics 2020;36:1453–9. 31665262 10.1093/bioinformatics/btz779

[32] Pires DE, Ascher DB. mCSM-AB: a web server for predicting antibody-antigen affinity changes upon mutation with graph-based signatures. Nucleic Acids Res 2016;44:W469–73. 27216816 10.1093/nar/gkw458 PMC4987957

[33] Pires DE, Ascher DB. CSM-lig: a web server for assessing and comparing protein-small molecule affinities. *Nucleic Acids Re*s 2016;44:W557–61. 27151202 10.1093/nar/gkw390 PMC4987933

[34] Pires DE, Blundell TL, Ascher DB. mCSM-lig: quantifying the effects of mutations on protein-small molecule affinity in genetic disease and emergence of drug resistance. Sci Rep 2016;6:29575. 27384129 10.1038/srep29575 PMC4935856

[35] Pires DEV, Ascher DB. mCSM-NA: predicting the effects of mutations on protein-nucleic acids interactions. Nucleic Acids Res 2017;45:W241–6. 28383703 10.1093/nar/gkx236 PMC5570212

[36] Rodrigues CHM, Myung Y, Pires DEV, et al. mCSM-PPI2: predicting the effects of mutations on protein-protein interactions. Nucleic Acids Res 2019;47:W338–44. 31114883 10.1093/nar/gkz383 PMC6602427

[37] Portelli S, Myung Y, Furnham N, et al. Prediction of rifampicin resistance beyond the RRDR using structure-based machine learning approaches. Sci Rep 2020;10:18120. 33093532 10.1038/s41598-020-74648-y PMC7581776

[38] Pires DE, Blundell TL, Ascher DB. pkCSM: predicting small-molecule pharmacokinetic and toxicity properties using graph-based signatures. J Med Chem 2015;58:4066–72. 25860834 10.1021/acs.jmedchem.5b00104 PMC4434528

[39] Al-Jarf R, de Sa AGC, Pires DEV, et al. pdCSM-cancer: using graph-based signatures to identify small molecules with anticancer properties. J Chem Inf Model 2021;61:3314–22. 34213323 10.1021/acs.jcim.1c00168 PMC8317153

[40] Rodrigues CHM, Pires DEV, Ascher DB. pdCSM-PPI: using graph-based signatures to identify protein-protein interaction inhibitors. J Chem Inf Model 2021;61:5438–45. 34719929 10.1021/acs.jcim.1c01135

[41] Velloso JPL, Ascher DB, Pires DEV. pdCSM-GPCR: predicting potent GPCR ligands with graph-based signatures. Bioinform Adv 2021;1:vbab031. 34901870 10.1093/bioadv/vbab031 PMC8651072

[42] Landrum G. RDKit: Open-Source Cheminformatics Software. (2016), URL http://www.rdkit.org/, https://github.com/rdkit/rdkit 2016.

[43] Pedregosa F, Varoquaux G, Gramfort A, et al. Scikit-learn: machine learning in python. J Mach Learn Res 2011;12:2825–30.

[44] Benesty J, Chen J, Huang Y, et al. Pearson correlation coefficient. Noise Reduction in Speech Processing. Berlin, Heidelberg: Springer Berlin Heidelberg, 2009, 1–4.

[45] Pires DEV, Ascher DB. mycoCSM: using graph-based signatures to identify safe potent hits against mycobacteria. J Chem Inf Model 2020;60:3450–6. 32615035 10.1021/acs.jcim.0c00362

[46] Vafaie H, Imam IF. Feature selection methods: genetic algorithms vs. greedy-like search. In Proceedings of the international conference on fuzzy and intelligent control systems. 1994;51:p. 28.

[47] Polat H, Danaei Mehr H, Cetin A. Diagnosis of chronic kidney disease based on support vector machine by feature selection methods. J Med Syst 2017;41:55. 28243816 10.1007/s10916-017-0703-x

[48] Ranjan A, Singh VP, Mishra RB, et al. Sentence polarity detection using stepwise greedy correlation based feature selection and random forests: an fMRI study. J Neurolinguistics 2021;59:100985.

[49] Borgelt C, Meinl T, Berthold M. MoSS: a program for molecular substructure mining. Proceedings of the 1st international workshop on open source data mining: frequent pattern mining implementations. Chicago, IL: Association for Computing Machinery, 2005, 6–15.

[50] Hao G, Dong Q, Yang G. A comparative study on the constitutive properties of marketed pesticides. Mol Inform 2011;30:614–22. 27467161 10.1002/minf.201100020

[51] Clarke ED, Delaney JS. Physical and molecular properties of agrochemicals: an analysis of screen inputs, hits, leads, and products. CHIMIA Int J Chem 2003;57:731–4.

[52] Tice CM . Selecting the right compounds for screening: does Lipinski's rule of 5 for pharmaceuticals apply to agrochemicals? Pest Manag Sci 2001;57:3–16. 11455629 10.1002/1526-4998(200101)57:1<3::AID-PS269>3.0.CO;2-6

[53] Gandy MN, Corral MG, Mylne JS, et al. An interactive database to explore herbicide physicochemical properties. Org Biomol Chem 2015;13:5586–90. 25895669 10.1039/c5ob00469a

[54] Lipinski CA, Lombardo F, Dominy BW, et al. Experimental and computational approaches to estimate solubility and permeability in drug discovery and development settings. Adv Drug Deliv Rev 1997;23:3–25. 10.1016/s0169-409x(00)00129-0 11259830

[55] Seralini G-E, Jungers G. Toxic compounds in herbicides without glyphosate. Food Chem Toxicol 2020;146:111770. 33027613 10.1016/j.fct.2020.111770

[56] Cheng F, Li W, Zhou Y, et al. admetSAR: a comprehensive source and free tool for assessment of chemical ADMET properties. J Chem Inf Model 2012;52:3099–105. 23092397 10.1021/ci300367a

[57] Gandhi K, Khan S, Patrikar M, et al. Exposure risk and environmental impacts of glyphosate: highlights on the toxicity of herbicide co-formulants. Environ Challenges 2021;4:100149.

