# Supplementary material for: cropCSM: designing safe and potent herbicides with graph-based signatures
Source: Brief Bioinform. 2022 Feb 24;23(2):bbac042. doi: 10.1093/bib/bbac042 (PMC9155605; doi:10.1093/bib/bbac042)
Supplement: cropCSM-Supplementary_bbac042 [file cropcsm-supplementary_bbac042.pdf]

SUPPORTING INFORMATION

cropCSM: designing safe and potent herbicides with graph-based signatures

Douglas E. V. Pires[1,2,3,4,*], Keith A. Stubbs[5], Joshua S. Mylne[5], David B. Ascher[1,2,3,6,*]

[1] Computational Biology and Clinical Informatics, Baker Heart and Diabetes Institute, Melbourne VIC 3004

[2] Department of Biochemistry and Molecular Biology, University of Melbourne, Melbourne, VIC 3010

[3] Computational and Systems Biology, Bio21 Institute, University of Melbourne, 30 Flemington Rd, Parkville VIC 3052

[4] School of Computing and Information Systems, University of Melbourne, Melbourne, VIC 3010

[5] The University of Western Australia, School of Molecular Sciences, 35 Stirling Highway, Crawley, Perth 6009, Australia

[6] Department of Biochemistry, University of Cambridge, 80 Tennis Ct Rd, Cambridge CB2 1GA

*To whom correspondence should be addressed D.B.A. Tel: +61 90354794; Email: david.ascher@unimelb.edu.au. Correspondence may also be addressed to D.E.V.P. douglas.pires@unimelb.edu.au.

FIGURES

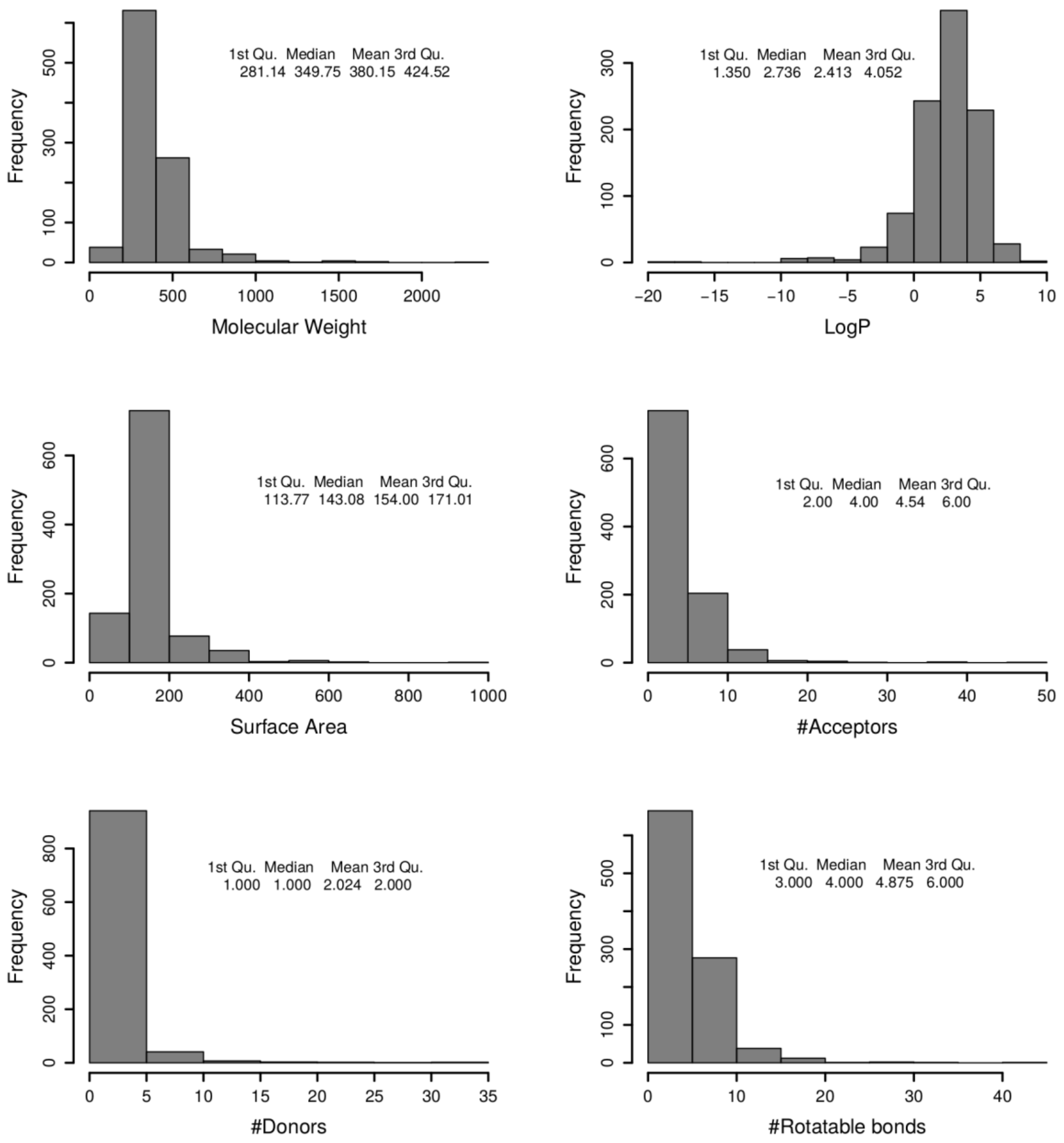

Figure S1. Property distribution for molecules with herbicidal activity. Herbicides seem to present similar properties, although slightly more lenient, to orally bioavailable drugs adherent to the Lipinski's Rule of 5.

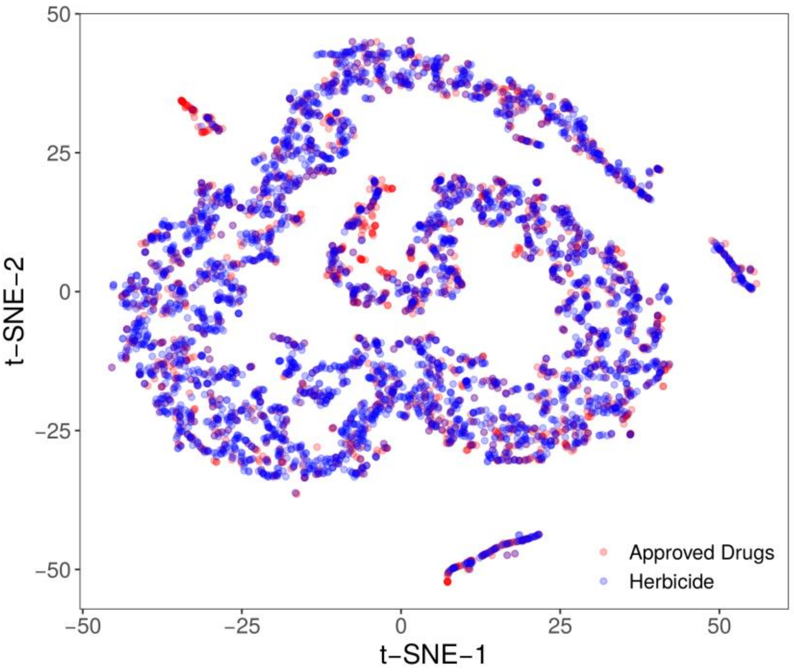

Figure S2. t-distributed stochastic neighbor embedding (t-SNE) plot comparing common physicochemical properties of approved drugs and herbicides. No distinction was identified between these two groups of molecules for a range of different parameters (t-SNE perplexity was systematically assessed). The t-SNE plot was generated using the R package 'tsne' with default parameters.

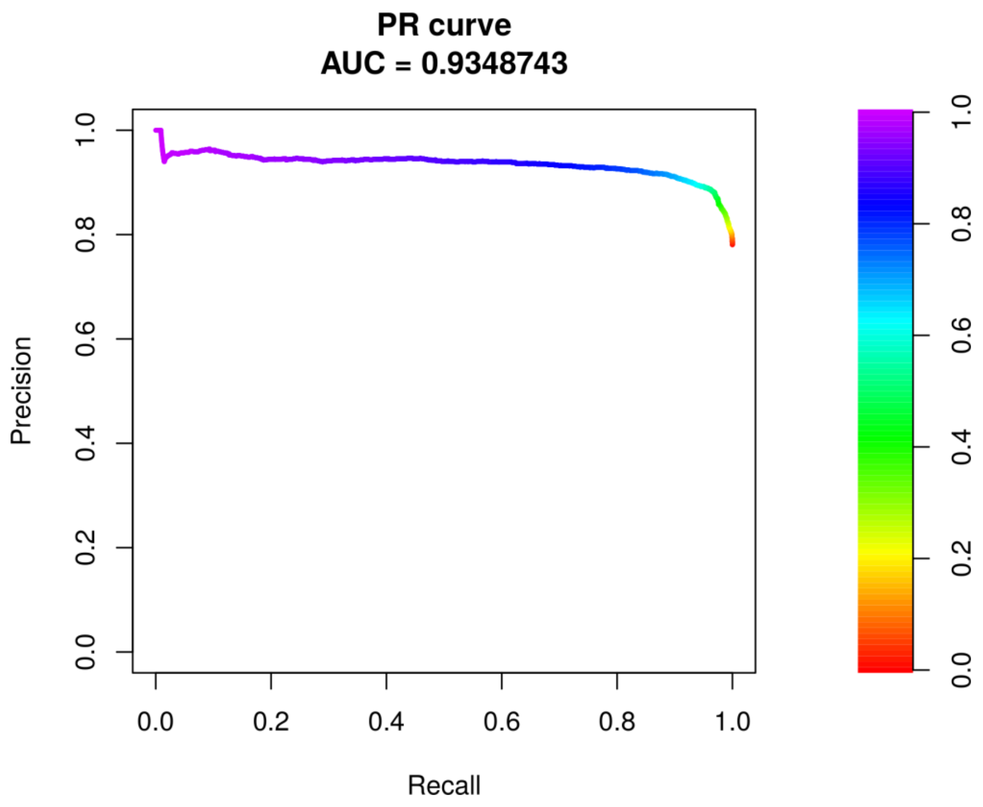

Figure S3. Precision-recall curve for the herbicidal activity predictor. cropCSM achieved and PR AUC of 0.93 demonstrating balanced performance.

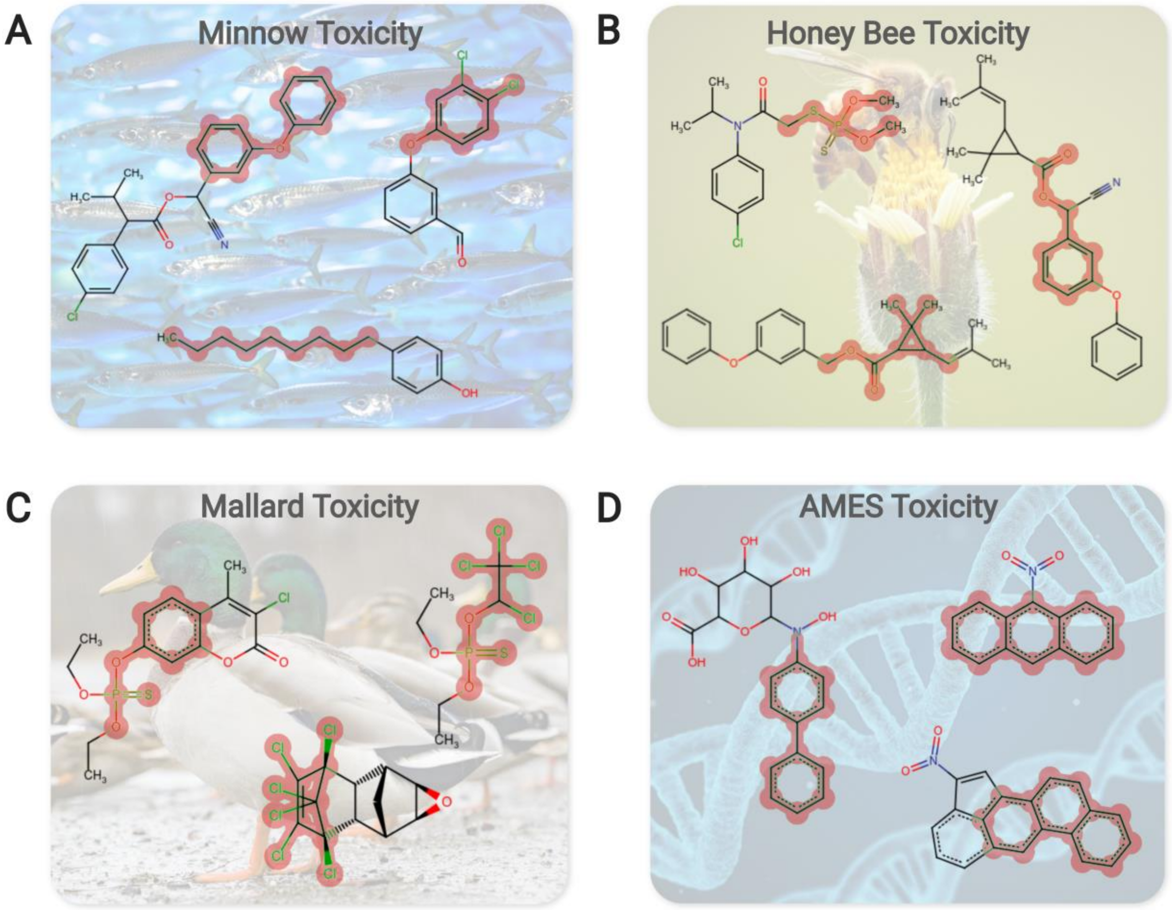

Figure S4. Substructure mining for toxicity predictors. The figure depicts common enriched substructures in compounds deemed toxic for flathead minnow (A), honey bee (B), mallard (C) and based on AMES toxicity/mutagenicity (D).

TABLES

Table S1. Details on cropCSM models and hyperparameters used.

**Predictive model**	**Learning Algorithm**	**Hyperparameters**
Herbicidal Activity	Random Forest	*n_estimators=300, random_state=1*
Minnow Toxicity (LC50)	Extra Trees	*n_estimators=300, random_state=1*
Oral Rat Chronic Toxicity	Random Forest	*n_estimators=300, random_state=1*
Oral Rat Acute Toxicity (LD50)	Extra Trees	*n_estimators=300, random_state=1*
Honey Bee Toxicity	XGBoost	*n_estimators=300, random_state=1*
Avian Toxicity	Gradient Boosting	*n_estimators=300, random_state=1*
AMES Toxicity	Extra Trees	*n_estimators=300, random_state=1*
